# Handbuch Der Medicinischen Klinik

**Published:** 1848-01

**Authors:** 


*Handbuch der Medicinischen Klinik*, verfasst von Dr. C. Canstatt, ordentlichem Professor der Medicin an der Universität zu Erlangen und Mitgliede mehrerer gelehrter Gesellschaften. Zweite vermehrte Auflage. Zweiter Band. 8vo. S. 1098. Erlangen, 1847.

The receipt of this second and last volume of M. Canstatt's work on "Clinical Medicine, conveyed to us the gratifying information, that the laborious and able author is not dead, as we announced in our number for October, 1847, when noticing the other volumes of his extensive work. With regret, however, we learn from the *Vorwort*, dated at Pisa in December, 1846, that since the winter of 1845, he has been a heavy sufferer, (*ein schwer Leidender,*) and compelled to visit a more southern region in the hope of restoration to health. This circumstance he mentions, not—as he remarks—to obtain credit for the mode in which the volume is executed,—but under his necessary want of books of reference, as an excuse for deficiencies, which might otherwise have been supplied. It is, however, well fitted to take its place with the rest of the series; and had the author not drawn attention to the subject, few of his readers—we will venture to say—would have discovered any difference. It embraces the *exanthematous diseases—malarious diseases*, in which he includes intermittent and remittent fever, yellow fever, oriental cholera, plague and dysentery—*typhus—rheums and catarrhs*, including rheumatism, influenza, hooping cough,—*heat diseases*, comprising bilious fever, sporadic cholera,—*diseases from animal poisons*, including glanders and worms, rabies canina, pustula maligna—*poisons—diseases of development*, including diseases of the new-born, of puberty, of the childbed state—*chronic pests*, including syphilis, gonorrhœa, lepra, plica; and *constitutional dyscrasies*, including scurvy, purpura hæmorrhagica, icterus, urodialysis, gout, hemorrhoids, tuberculosis and scrophulosis, rickets, osteomalacia of adults, and cancer,—a somewhat singular classification, it must be admitted.

Although this second volume, which—*more Germanico*, appears long after the third and fourth—completes the consideration of individual diseases, we find we have still to wait—before the work can be bound—for the Index—*Sachregister*—which is promised in four weeks from the publication of the volume before us.

Of the character of the work of M. Canstatt, we gave our opinion in the number of the "Examiner" already referred to. We can recommend it to those who are acquainted with the German language. It contains a vast amount of information, and is free from the over-speculative fault of many of the productions of his countrymen on the same and similar subjects.

